# Translation, cultural adaptation and validation of the Chinese Multimorbidity Treatment Burden Questionnaire(C-MTBQ): a study of older hospital patients

**DOI:** 10.1186/s12955-020-01395-z

**Published:** 2020-06-22

**Authors:** Liyuan Dou, Juan Huang, Polly Duncan, Lixiang Guo

**Affiliations:** 1 School of Nursing and Health, Zhengzhou University, No.101, Kexue Road, Zhengzhou, Henan Province 450000 People’s Republic of China; 2 Party Secretary Office, People’s Hospital of Zhengzhou, Zhengzhou, Henan Province 450000 People’s Republic of China; 3 Centre for Academic Primary Care, University of Bristol, Bristol, BS6 6HL UK

**Keywords:** Elderly, Hospital, Treatment burden, Reliability, Validity, Multimorbidity

## Abstract

**Background:**

Due to an ageing population, multimorbidity is becoming more common. Treatment burden (the effort required of patients to look after their health and the impact this has on their wellbeing) is prevalent in patients with multimorbidity. The Multimorbidity Treatment Burden Questionnaire (MTBQ) is a patient-reported outcome measure of treatment burden that has been validated amongst patients with multimorbidity in the UK. The aim of this study was to translate and culturally adapt the MTBQ into Chinese and to assess its reliability and validity in elderly patients with multimorbidity in hospital.

**Methods:**

The original English version of the MTBQ was translated into Chinese using Brislin’s model of cross-culture translation. The C-MTBQ was piloted on a sample of 30 elderly patients with multimorbidity prior to being completed by 156 Chinese elderly patients with multimorbidity recruited from a hospital in Zhengzhou, China. We examined the proportion of missing data, the distribution of responses and floor and ceiling effects for each question. Factor analysis, Cronbach’s alpha, intraclass coefficient and Spearman’s rank correlations assessed dimensional structure, internal consistency reliability, test-retest reliability and criterion validity, respectively.

**Results:**

The average age of the respondents was 73.5 years (range 60–99 years). The median C-MTBQ global score was 20.8 (interquartile range 12.5–29.2). Significant floor effects were seen for all items. Factor analysis supported a three-factor structure. The C-MTBQ had high internal consistency (Cronbach’s alpha coefficient, 0.76) and test-retest reliability (the intraclass correlation coefficient, 0.944), the correlations between every item and global scores scored > 0.4. The scale content validity index(S-CVI) was 0.89, and the item level content validity index(I-CVI)was 0.83 ~ 1.00. The criterion validity was 0.875.

**Conclusion:**

The Chinese version of MTBQ showed satisfactory reliability and validity in elderly patients with multimorbidity, and could be used as a tool to measure treatment burden of elderly patients with multimorbidity in hospital.

## Background

Due to an ageing population, multimorbidity (two or more long-term conditions existing in one individual [1]) is increasing [2]. Studies have shown that the prevalence of multimorbidity in the elderly (≥ 65 years) is greater than 60%, and more than half of people with multimorbidity have three or more long term conditions [3]. Patients with multimorbidity experience higher disease burden than patients with single conditions and are at increased risk of high treatment burden- the effort required of patients to look after their health and the impact this has on their general wellbeing (e.g. attending multiple appointments with different health professionals, taking medicines at different times in the day) [4, 5]. Studies have shown that high treatment burden is associated with reduced quality of life and adherence to treatment [6, 7], high hospitalization rates and high mortality [8].

There is a lack of research investigating treatment burden-for-patients with multimorbidity in China. Having a validated measure of treatment burden is essential to improving understanding of factors associated with high and low treatment burden, and to testing interventions that aim to reduce treatment burden. There is one existing generic measure of treatment burden that has been translated into Chinese, known as the Chinese Treatment Burden Questionnaire (C-TBQ). This measure was validated in a younger study population (median age 62 years). It is also a longer questionnaire to complete with 15 questions.

The multimorbidity treatment burden questionnaire (MTBQ) was developed and validated in the UK to assess treatment burden for patients with multimorbidity. Based on the treatment burden framework developed by Eton et al. [4] in 2012, the MTBQ is short, simple, and easy to use [9]. It was validated in over 1500 older patients (mean age 71 years) with multimorbidity (≥ 3 long term conditions) and demonstrated good content validity, construct validity, reliability and responsiveness. The MTBQ has been translated into Danish and used in a population health survey, in which it showed good reliability and validity [10].

The purpose of this study was to translate and culturally adapt the MTBQ into Chinese and to test the psychometric properties of the questionnaire among Chinese-speaking elderly patients with multimorbidity in hospital.

## Method

### Participants

The data was collected from one hospital in Zhengzhou City, Henan province, China between August and October 2019. Patients were invited to take part if they met the following inclusion criteria: (1) complete hospital records and diagnosis at time of discharge; (2) two or more long term conditions; (3) age ≥ 60 years. Patients were excluded if they had a serious physical or mental illness. The sample size of 156 was calculated using the Consensus-based Standards for the Selection of Health Measurement Instruments (COSMIN) [11]. This guideline advises 5–10 participants per item of the questionnaire. We slightly increased the sample size to account for incorrect filling of questionnaires.

### Questionnaires

The Multimorbidity Treatment Burden Questionnaire (MTBQ) has 10 questions and three additional optional questions (questions which did not apply in a UK context but may apply to other populations). For the C-MTBQ, -these three additional questions were included. For each question, answers are ranked using a 5-point Likert scale, ranging from 0 (does not apply or not difficult),1 (a little difficult), 2(quite difficult), 3(very difficult) and 4 (extremely difficult). The global MTBQ score is calculated as the mean score, multiplied by 25, giving a score of 0 to 100. Global treatment burden scores can be categorized as: no-treatment burden (0), low treatment burden (< 10), medium treatment burden (10–22), high treatment burden (≥22).

The Treatment Burden Questionnaire (TBQ) was originally developed by Tran [12] and measures the perceived treatment burden of patients with long term conditions. It has been translated into many countries [12, 13]. It includes 15 questions and four dimensions: medication(1A ~ 1D), seeing doctors and subsequent visit (2A ~ 2E), medical related and lifestyle(3A ~ 3E) and health problem(4A), A 10-point Likert scale is used, ranging from 0 (no burden) to 10 (high burden). The global score of the TBQ is calculated as the sum of the answers to each item and ranges from 0 to 150, with higher scores indicating a higher level of treatment burden.

### Translation process

Researchers followed the Brislin model to translate the MTBQ into Chinese [14]. Step 1: The MTBQ was independently translated into Chinese by two researchers with Chinese linguistic backgrounds(T1, T2). T1 has a Master’s degree and is medically trained and T2 has a postgraduate degree in English with no medical training. Step 2: A third researcher, who has a Master’s degree in nursing and 6 years of IELTS training experience, reviewed and synthesized the translated versions created by T1 and T2, referring to the original English version. Step 3: two bilingual translators(One is a Master’s degree student in English, the other is a Nursing PhD)back-translated the synthesized version into English to highlight conceptual errors in the translations. Step 4: All translators and team members compared the original MTBQ and the two back-translation versions to form a comprehensive back-translation version to confirm accuracy [15]. The comprehensive back-translation was reviewed by the original MTBQ author(PD), and minor revisions were made until the comprehensive back-translation version and the original questionnaire had a semantic consistency rate of over 90%.

### Cross-cultural adaptation

Six experts conducted the cross-cultural adaptation: two general medical doctors; two hospital nurses who specialize in long-term conditions; a nurse who specializes in care of the elderly and the director of the care of the elderly hospital department.

They first evaluated the Chinese version of MTBQ in terms of accuracy, simplicity of the text, grammar, use of proper terms and syntax. The cultural relevance (language clarity, language habits, cultural background conformity and content relevance) and content validity were evaluated using a 4-point rating scale ranging from 1 (not relevant) to 4 (very relevant) to ensure the cultural applicability and content equivalence of the questionnaire [16]. The content validity index (CVI) (the proportion of questions rated by experts as either 3 or 4) was calculated.

### Piloting

The final version of the MTBQ was piloted in 30 elderly patients who met the inclusion criteria. We found that they could understand the items easily and that they required an average of 5 minutes to finish the questionnaire.

### Questionnaire administration

The questionnaires were administered face-to-face by three post-graduate students who were trained by the research team. Participants were identified by nursing staff working on the hospital wards. Data from the patient’s hospital records was collected with written consent.

### Statistical analysis

Data were analysed using SPSS version 21.0. Descriptive statistics were generated to describe the participants’ characteristics. The distribution of responses for each question, proportion of missing data, proportion of ‘does not apply’ responses and floor and ceiling effects were examined. An exploratory factor analysis (EFA) was conducted to evaluate the dimensionality of the questionnaire [17], and the number of extracted factors was determined using the principal-component analysis (PCA) and varimax rotation. Factor loadings (λ) > 0.40 or < − 0.40 were considered acceptable [18].

To assess internal consistency reliability, we examined the inter-item correlation matrix and calculated Cronbach’s alpha (0.7–0.95 was deemed acceptable) [11, 19]. Test-retest reliability was assessed by comparing the total C-MTBQ scores in a subset of patients who completed the questionnaire on both day 0 and day 14. The intraclass correlation coefficient (ICC) was calculated and interpreted as follows: excellent (> 0.8), good (0.61–0.80), moderate (0.41–0.60), fair (0.21–0.40) and poor (≤0.20) [20]. To assess criterion validity, we examined the relationships between C-MTBQ and the Chinese version of the TBQ.

We examined the distribution of scores for each question. Ceiling and floor effects were considered to be present if more than 15% of respondents achieved the lowest (0) or highest (4) score, respectively.

### Ethical approval

The study was approved by the ethics committee of People’s Hospital of Zhengzhou. Written consent was taken for all the participants.

## Results

### Translation and cultural adaptation

“Health professionals” (questions 6, 7, and 8) was replaced by the traditional Chinese word for “medical staff” as the experts felt this was, the more commonly used expression. During interviews with participants, for the “appointments” in questions 6 and 8, the participants reports that there was no need to make an appointment to see a doctor in hospital mostly, so we removed the word “appointments”. The item CVIs ranged from 0.83 ~ 1.00, while the scale CVI was 0.89, indicating good content validity of the C-MTBQ.

### Description of sample

One hundred and fifty-six participants completed the study. There characteristics are shown in Table 1. The average age was 73.5 years (60–99 years) with slightly more males (54.5%). Less than a fifth had a college education. Two thirds of patients had three or more long-term conditions.

**Table 1:** Sample characteristics (*n* = 156)

Characteristics	N	%
Gender		
Male	85	54.5
Female	71	45.5
Age(years)		
60 ~ 70	63	40.4
70 ~ 80	46	29.5
80 ~ 90	40	25.6
≥ 90	7	4.5
Marital status		
married	132	84.6
Single/divorced/widowed	24	15.4
Education Level		
No formal/Primary education	39	25.0
Secondary education	91	58.3
Tertiary education	26	16.7
Household income per month(Yuan)		
< 1000	30	19.2
1000 ~ 3000	41	26.3
3000 ~ 5000	53	34.0
≥ 5000	32	20.5
Living Status		
Living alone	22	14.1
Living with others	134	85.9
Health insurance		
Town medical insurance	115	73.7
New Rural Cooperative	27	17.3
Public medical care	13	8.3
Others	1	0.6
Long-term conditions		
Cardiovascular Disease	149	95.5
Stroke/Transient Ichaemic attack	104	66.7
Diabetes	81	51.9
Chronic kidney disease	34	21.8
Chronic obstructive culnar disease or asthma	20	12.8
Atrial fibrillation	23	14.7
Depression	7	4.5
Joint disease	31	19.9
Heart failure	14	9.0
Number of chronic diseases		
2	53	34.0
3	72	46.2
4	19	12.2
≥ 5	12	7.7

### The descriptive statistics of the C-MTBQ

The proportion of missing data for each question was 0% (see Table 2). For the optional question about “Getting help from community health services (eg, physical therapy, health services provided by community nurses, etc.)”, 64% of patients responded “does not apply”. As this was greater than 40%, this question was removed from the questionnaire. High floor effects (the proportion of participants who responded ‘not difficult’ or ‘does not apply’) were found for all questions. The range of skewness was between 0.453 and 2.093, and the range of kurtosis was between 0.040 and 3.721, indicating that the items of the C-MTBQ were non-normally distributed. The global C-MTBQ scores were skewed and varied from 2 to 60. None of participants had a global C-MTBQ score of 0, and no treatment burden(0), low treatment burden(< 10), medium treatment burden(10–22) and high treatment burden (≥22) accounted for 0, 14.7, 39.7 and 45.5%, respectively. The median C-MTBQ total score was 20.8 (interquartile range 12.5–29.2).

**Table 2:** Response to the C-MTBQ (*n* = 156)

Item, Description of item	N	Not difficult n(n/N%)	A little difficult n(n/N%)	Quite difficult n(n/N%)	Very difficult n(n/N%)	Extremely difficult n(n/N%)	Dose not applyn(n/N%)
1. Taking lots of medications	156	73(47)	60(39)	20(13)	2(1)	1(1)	0(0)
2. Remembering how and when to take medication	156	90(58)	44(28)	19(12)	1(1)	0(0)	2(1)
3. paying for medications and treatment	156	59(38)	31(20)	20(13)	32(21)	14(9)	0(0)
4. take medicine regularly	156	45(29)	55(35)	30(19)	4(3)	1(1)	21(14)
5.Monitoring your medical conditions (e.g. checking your blood pressure or blood sugar, monitoring your symptoms etc.)	156	34(22)	46(30)	29(19)	20(13)	2(1)	25(16)
6. To see a doctor about a health issue	156	59(38)	62(40)	27(17)	4(3)	0(0)	4(3)
7. Go to see different doctors	156	39(25)	75(48)	24(15)	3(2)	0(0)	15(10)
8. getting time off work, arranging transport etc. to see doctors	156	64(41)	44(28)	16(10)	2(1)	0(0)	30(19)
9. Getting health care in the evenings and at weekends	156	98(63)	26(17)	4(3)	0(0)	0(0)	28(18)
10. Getting help from community services (e.g. physiotherapy, district nurses etc.)	156	23(15)	15(10)	11(7)	6(4)	1(1)	100(64)
11.Obtaining clear and up-to-date information about your condition	156	29(19)	65(42)	32(21)	26(17)	4(3)	0(0)
12.Making recommended lifestyle changes (e.g. diet and exercise etc.)	156	30(19)	65(42)	45(29)	10(6)	0(0)	6(4)
13.Must rely on support from family members and friends	156	11(7)	72(46)	25(16)	0(0)	1(1)	47(30)

### Factor analysis

**Fig. 1:**
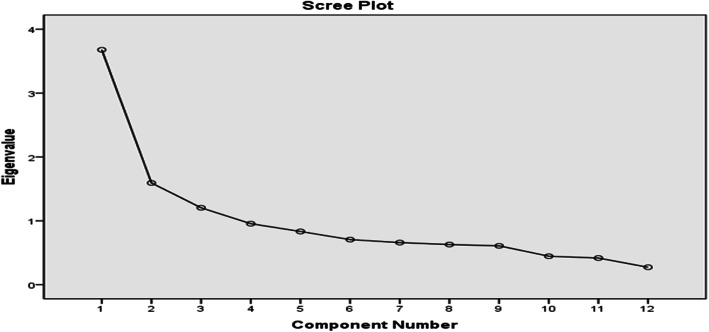
Scree plot of C-MTBQ

The Kaiser-Meyer-Olkin (KMO) measure of sampling adequacy(0.776) and the Bartlett test of sphericity (*p* < 0.001) showed that factor analysis of the data was appropriate [21, 22]. The scree plot is shown in Fig. 1. Three common factors were obtained, which explained the total variance of 54%, loadings of all items ranged from 0.505 to 0.816. The eigenvalue of the three factors were -2.494, 2.022 and 1.960, respectively. The original English MTBQ had only one dimension. According to the content characteristics, factor 1 (1,2,6,7) was named as the medication and treatment dimension (4 items); factor 2 (3,4,8,9,13) was named as the medical related dimension (5 items), and factor 3(5、11、12) was named as the daily self-health management dimension. The three dimensions represented are shown in Table 3.

**Table 3:** Factor analysis, internal consistency of C-MTBQ

Item	medication and treatment	medical related	daily self-health management	Cronbach’s Alpha if Item Deleted	Corrected item-total correlation
1	**0.816**	0.081	0.063	0.736	0.527
2.	**0.798**	−0.089	0.254	0.735	0.536
6	**0.641**	0.296	0.341	0.715	0.695
7.	**0.680**	0.314	−0.07	0.739	0.501
3.	−0.226	**0.708**	0.119	0.778	0.455
4.	0.312	**0.561**	0.415	0.713	0.701
8	0.202	**0.619**	0.068	0.738	0.514
9	0.240	**0.568**	−0.047	0.748	0.403
13	0.086	**0.505**	0.453	0.731	0.573
5	0.247	−0.068	**0.586**	0.751	0.485
11	0.033	0.171	**0.716**	0.736	0.564
12	0.024	0.106	**0.718**	0.741	0.499
Eigenvalue	**2.494**	**2.022**	**1.960**		
% of Variance Explained (rotation solution)	**20.780**	**16.850**	**16.337**		
Cumulative % of Variance Explained (rotation solution)	**20.780**	**37.629**	**53.966**		

A value of 0.40 or greater for the factor loadings was regarded as acceptable.

### Reliability

The Cronbach’s alpha and the corrected item-total correlations of all 12 questions are shown in Table 3. Item-total correlations ranged from 0.403 (item 9) to 0.701 (item 4), and all items met the recommended minimum of 0.20. Removing any item of the C-MTBQ didn’t result in severely changing in the value of Cronbach’s alpha. The internal consistency coefficient for the total score of the C-MTBQ were showed with a Cronbach’s alpha of 0.755. Thirty of the 156 participants were randomly selected to evaluate the test-retest reliability. The mean scores of the first and second measurements were 19.79 (interquartile range 19.79–29.17) and 21.00 (interquartile range 14.75–25.50), respectively. The test-retest reliability was satisfactory with an ICC of 0.944.

### Criterion validity

Correlations of C-MTBQ with TBQ and TBQ subscales scores are displayed in Table 4. The results suggested moderate to high correlations of C-MTBQ with TBQ score (r = 0.875) and TBQ subscales scores (r: 0.495 ~ 0.740).

**Table 4:** Correlation (r) for the C-TBQ and C-TBQ subscales with the C-MTBQ of the Constant-Murley score

Criterions	C-TBQ -medication	C-TBQ-seeing doctors and subsequent visit	C-TBQ-medical related and lifestyle	C-TBQ-health problem	TBQ
C-MTBQ	0.726	0.589	0.740	0.495	0.875

*C-MTBQ* The Chinese version of the Multimorbidity Treatment Burden Questionnaire, *C-TBQ* The Chinese version of the Treatment Burden Questionnaire

## Discussion

In this study we have translated, culturally adapted and validated a 12-item questionnaire, named the Chinese Multimorbidity Treatment Burden Questionnaire (C-MTBQ). We followed the standard forward-backward translation process and examined the psychometric properties of the C-MTBQ in hospitalized Chinese elderly patients with multimorbidity. The C-MTBQ demonstrated good content validity, internal consistency reliability, test-retest reliability and criterion validity. A three-factor structure was found, which is different to the one factor structure of the original MTBQ.

The question 10 “Getting help from community health services (eg, physical therapy, health services provided by community nurses, etc.)” was deleted, because 64% of patients responded “does not apply”. This may be due to differences between community health services in the UK and China, with primary care being less well developed in China and patients preferring to seek treatment from hospitals [23, 24].

The median C-MTBQ total score was 20.8, and the median C-TBQ score was 16. It is difficult to draw comparisons since the global score for each questionnaire is calculated differently [25]. All the questions have high floor effects, which were similar to the C-TBQ. This may be in part due to the sample frame as all the participants were elderly, unemployed or retired and hence they had time to remember how and when to take medications and to monitor their medical conditions. Also they did not need to take time off work to see doctors. Further studies involving younger patient populations, particularly those with busy work patterns may reveal a different pattern of scores. A lack of a ceiling effect indicates that the C-MTBQ may be better for monitoring deterioration in treatment burden.

According to the results of factor analysis, all of the 12 questions of the C-MTBQ were grouped into three factors, and factor loadings reached the criteria of 0.40. By comparison, the original MTBQ was unidimensional. This may have potential implications for instrument scoring. There are several explanations for this. Firstly, item 10 was deleted from the C-MTBQ. Secondly, the sample size for the C-MTBQ was small compared to the original English questionnaire which was validated in 1500 patients with multimorbidity [9, 26]. Thirdly, the participants of the original questionnaire were ≥ 18 years, participants of this research were aged ≥60 years. Lastly, there are important cultural differences between the UK and China and this is likely to impact on perceived treatment burden [27]. In terms of criterion validity, the C-MTBQ correlated well with TBQ score (r = 0.875), which indicated that the C-MTBQ had a good criterion validity.

For the Internal consistency, the results showed a little lower internal consistency with a Cronbach’s alpha of 0.755 than the value validated in original MTBQ(0.83) [9], indicating good reliability. The test-retest reliability coefficient of our questionnaire was 0.944, which showed that the questionnaire had time consistency.

The MTBQ uses simple language and is suitable for elderly people to complete. The average time to complete the questionnaire was about 5 minutes, which was shorter than C-TBQ. The questionnaire has clear and detailed instructions. For investigators using the questionnaire, there are clear instructions on calculating, reporting and interpreting global MTBQ scores. However, there are also limitations. Firstly, the sample size was relatively small. Secondly, convenience sampling method were used and the samples were all from only one hospital in Zhengzhou, China, which may make it difficult to generalize the findings to a wider population in China. Moreover, this research only focused on older adults and the results may not be generalizable to younger people. Further research is needed to validate the C-MTBQ amongst younger people living in other provinces of China.

## Conclusion

The Chinese version of multimorbidity treatment burden questionnaire (C-MTBQ) had good reliability and validity. It can be used as a patient-reported outcome measure to assess the treatment burden in Chinese-speaking elderly patients with multimorbidity in hospital. 

## Data Availability

Please contact author for data requests.

## Abbreviations

MTBQ: Multimorbidity treatment burden questionnaire
TBQ: Treatment burden questionnaire
COSMIN: The Consensus-based Standards for the Selection of Health Measurement Instruments
ICC: Intra-class correlation coefficient
EFA: Exploratory factor analysis
C-MTBQ: The Chinese vesion of Multimorbidity treatment burden questionnaire
PCA: Principal-component analysis
SD: Standard deviation
CVI: Content validity index
S-CVI: Scale content validity index
I-CVI: Item content validity index
KMO: Kaiser-Meyer-Olkin
PROM: Patient Reported Outcome Measures
